# Evaluation of the combined predictive value of multiple indicators based on diaphragmatic ultrasound using logistic regression and ROC curve in weaning from mechanical ventilation in pediatric patients

**DOI:** 10.3389/fped.2024.1344709

**Published:** 2024-07-04

**Authors:** Hejia Ge, Ailian Zhang, Yiqun Teng, Li Hu

**Affiliations:** ^1^ Department of Pediatrics, The Second Affiliated Hospital of Jiaxing University, Jiaxing, China; ^2^ Department of Anesthesiology, The Second Affiliated Hospital of Jiaxing University, Jiaxing, China

**Keywords:** mechanical ventilation, diaphragm ultrasonography, weaning, logistic regression, ROC curve

## Abstract

**Background:**

Conventional single indicators have low sensitivity and specificity for predicting weaning from mechanical ventilation in pediatric patients, necessitating the establishment of a combined prediction model for predicting weaning outcomes.

**Objectives:**

To explore the combined predictive value of PaO_2_/FiO_2_ Ratio (P/F ratio), diaphragm excursion-rapid shallow breathing index (DE-RSBI), diaphragm thickening fraction-rapid shallow breathing index (DTF-RSBI), and Pediatric Critical Illness Score (PCIS) in weaning from mechanical ventilation in pediatric patients.

**Methods:**

Sixty critically ill pneumonia pediatric patients requiring mechanical ventilation treatment from July 2022 to June 2023 at the Second Affiliated Hospital of Jiaxing University were selected. They all underwent a spontaneous breathing trial (SBT) and were divided into the weaning success group (42 cases) and weaning failure group (18 cases) based on the weaning outcome. Parameters including total duration of illness, mechanical ventilation duration, heart rate (HR), P/F ratio, diaphragm excursion (DE), DE-RSBI, diaphragm thickening fraction (DTF), DTF-RSBI, and PCIS were included in univariate and multivariate logistic regression analyses to determine independent factors affecting pediatric weaning success. Receiver operating characteristic (ROC) curves were plotted to evaluate the predictive value of P/F ratio, DE-RSBI, DTF-RSBI, PCIS alone or in combination for weaning success.

**Results:**

Comparing P/F ratio, DE, DE-RSBI, DTF, DTF-RSBI and PCIS, there were statistically significant differences (*P *< 0.05). Through collinearity analysis and binary logistic regression analysis,P/F ratio [OR* *=* *0.777, 95% CI (0.641,0.941)], DE-RSBI [OR* *=* *1.694, 95% CI (1.172, 2.447)], DTF-RSBI [OR* *=* *1.057, 95% CI (1.002, 1.114)], and PCIS [OR* *=* *0.661, 95% CI (0.445, 0.982)] were identified as independent factors affecting successful weaning(*P *< 0.05).The regression equation was: Logit*P* = 73.299–0.253 P/F ratio + 0.525DE-RSBI + 0.055DTF-RSBI-0.43PCIS.The sensitivity of the combined indicator Logit(*P*) in predicting successful weaning from mechanical ventilation in pediatric patients was 88.9%, with a specificity of 95.2% (optimal cutoff value of 0.511), and the area under the ROC curve (AUC) was 0.960 [95% CI (0.915, 1.000)]. The AUC of the combined prediction model for predicting pediatric weaning was greater than that of P/F ratio, DE-RSBI, DTF-RSBI and PCIS alone (*Z* values* *=* *9.129, 2.061, 2.075, 8.326, *P *< 0.05).

**Conclusions:**

In mechanically ventilated pediatric patients, the combined prediction model has better predictive value for weaning success compared to using P/F ratio, DE-RSBI, DTF-RSBI, or PCIS alone.

## Introduction

In the Pediatric Intensive Care Unit (PICU), mechanical ventilation is a commonly used life-support intervention for children with respiratory failure (1). However, prolonged mechanical ventilation can lead to pulmonary complications such as ventilator-associated pneumonia and lung injury (2, 3). Therefore, safely and timely weaning from mechanical ventilation poses a significant challenge for clinical pediatricians. In fact, the decision to wean involves considering multiple factors, including oxygenation status, respiratory drive, and respiratory muscle function.

Currently, decisions regarding weaning are primarily based on laboratory test results and clinical assessments, such as blood gas analysis and spontaneous breathing trials. In recent years, the use of diaphragmatic ultrasound has become increasingly widespread and is considered a non-invasive, real-time tool for assessing diaphragm function. Studies have shown that diaphragmatic ultrasound can predict the recovery of diaphragm function and weaning outcomes in patients (4). However, the sensitivity and specificity of single indicators for predicting weaning are less than ideal (5). Therefore, there is a clinical need for an effective, rapid, and straightforward method for predicting weaning.

The aim of this study is to apply logistic regression to construct a combined prediction model and investigate its clinical value in weaning prediction.

## Materials and methods

### Study population

Sixty pre-school-aged patients underging mechanical ventilation in the PICU at the Second Affiliated Hospital of Jiaxing University from July 2022 to June 2023 were selected for this study. They were categorized into the weaning success group and weaning failure group based on the outcome of the extubation process. Informed consent was obtained from all guardians. Inclusion criteria: Invasive mechanical ventilation for more than 48 h; Meeting the Berlin criteria for acute respiratory distress syndrome, with an P/F ratio <300 mm Hg (6). Exclusion criteria: Patients with nasal intubation, non-invasive ventilation, or tracheostomy; Unplanned extubation (including accidental or self-extubation); History of diaphragmatic paralysis, chest trauma, cervical spinal cord injury, neuromuscular junction disorders, pneumothorax, and mediastinal emphysema; End-stage cancer patients; Presence of mediastinal emphysema, pneumothorax, or closed chest drainage.

### Study methods

After significant improvement in the patient's condition, a Spontaneous Breathing Trial (SBT) was conducted using a low-pressure spontaneous breathing mode for weaning. The ventilation mode was changed to Pressure Support Ventilation (PSV), with a pressure support level ≤7 cm H_2_O with or without Positive End-expiratory Pressure (PEEP) ≤ 5 cm H_2_O. Oxygen concentration was maintained, and SBT was initiated after suctioning. The SBT duration was 120 min, and successful weaning was defined as the patient maintaining spontaneous breathing without the need for reintubation or non-invasive ventilation within 48 h after extubation. Weaning failure was defined as reintubation, advanced oxygen therapy, or death within 48 h after extubation. The criteria for SBT failure were based on the 2009 standards of the American Pediatric Critical Care Research Group (7).

### General information recording

Record general data for both groups, including age, duration of illness, duration of mechanical ventilation, heart rate (HR), and respiratory rate (RR), the specific situation of the weaning failure group were recorded; PaO_2_/FiO_2_ Ratio (P/F ratio):Arterial blood gas analysis was performed to obtain PaO_2_ and FiO_2_, and P/F ratio was calculated as PaO_2_/FiO_2_; Pediatric Critical Illness Score (PCIS):PCIS was assessed in preparation for weaning.

### Observation indicators and methods for diaphragmatic ultrasound before SBT

Diaphragmatic Excursion (DE): The patient's head was elevated by 20°–40°, and an edge ultrasound machine (Sonosite, USA) with a 10 MHz linear array probe was placed at the junction of the mid-clavicular line or anterior axillary line and the lower border of the rib cage. The liver was used as a window, and the probe was directed towards the head and back to visualize the lower third of the diaphragm. DE (cm) was calculated as the distance from the baseline to the diaphragm at the end of inhalation minus the distance at the end of exhalation.

Diaphragmatic Thickening Fraction (DTF): Using the M-mode, the right diaphragm was continuously observed in the 8–10 intercostal space in the right mid-axillary line. Diaphragmatic thickness at end-inspiration (DTi) and end-expiration (DTe) were measured, and DTF (%) was calculated as (DTi - DTe)/DTe * 100%. The average DTF was calculated over 3–5 respiratory cycles for each patient.

Diaphragmatic Excursion - Rapid Shallow Breathing Index (DE-RSBI) and Diaphragmatic Thickening Fraction - Rapid Shallow Breathing Index (DTF-RSBI): DE-RSBI was calculated as RR/DE, and DTF-RSBI was calculated as RR/DTF.

### Statistical methods

Statistical analysis was conducted using SPSS 21.0 (IBM, Armonk, NY, USA). Normally distributed continuous data were presented as means ± standard deviation. Independent sample *t*-tests were employed for group comparisons. Count data were expressed as frequencies and percentages, and group comparisons were performed using the *χ*^2^ test or Fisher's exact test.

Single-factor analysis was conducted for two groups. Indicators with statistically significant differences in single-factor analysis were treated as independent variables, and successful weaning was treated as the dependent variable in logistic regression. First, regression applicability conditions were verified (linearity between continuous independent variables and log-transformed dependent variables, and the presence of multicollinearity between independent variables). After verification, univariate receiver operating characteristic curves (ROC) were plotted for successful weaning, with the optimal cutoff value used as the threshold for binary classification. Subsequently, multivariate logistic stepwise regression was conducted to construct a joint prediction model (variable selection criteria set at entry = 0.10, removal = 0.15), with outliers removed and re-regression performed if necessary. Regression coefficient (b) testing, model goodness-of-fit testing using the Hosmer-Lemeshow test (*P* > 0.05 indicating good fit), and odds ratio (OR) analysis were conducted.

The Box-Tidwell test was utilized to assess the linear relationship between continuous univariate and log-transformed dependent variables. Linear regression was employed to ensure tolerance >0.1 or variance inflation factor <10, indicating no multicollinearity. Outliers were defined as values exceeding 3 times the standard deviation. The Wald test evaluated regression coefficients (b). The predictive value of weaning success was evaluated using Receiver Operating Characteristic (ROC) curves, with the area under the ROC curve (AUC) compared using the non-parametric Delong method. Statistical significance was set at *P* < 0.05.

## Results

### Univariate analysis of factors affecting weaning success and failure

This study included a total of 60 pediatric patients, among whom 18 experienced weaning failure. Weaning failure cases comprised: reconnected to mechanical ventilation after spontaneous breathing trial (*n* = 12), non-invasive ventilation within 48 h (*n* = 2), and reintubation within 48 h (*n* = 4). There were no statistically significant differences (*P *> 0.05) between the weaning success and failure groups in terms of disease duration, mechanical ventilation time, age, HR and MAC. However, significant differences were observed (*P *< 0.05) in P/F ratio, DE, DE-RSBI, DTF DTF-RSBI, and PCIS between the two groups, as shown in Table 1.

**Table 1:** Univariate analysis of factors affecting the success and failure of aircraft retrieval.

Group	Number	Duration ($\bar{x}\pm s$, days)	Mechanical ventilation time ($\bar{x}\pm s$, days)	Age ($\bar{x}\pm s$, years)	P/F ratio ($\bar{x}\pm s$, mm Hg)	HR ($\bar{x}\pm s$, 次/min)	MAC ($\bar{x}\pm s$, mm Hg)	DE (x±s, dm)	DE-RSBI [$\bar{x}\pm s$, beats/ (min·dm)]	DTF (x±s)	DTF-RSBI ($\bar{x}\pm s$, beats/min)	PCIS ($\bar{x}\pm s$, scores)
Weaning success group	42	10.86 ± 3.95	8.64 ± 3.28	3.64 ± 2.28	203.81 ± 10.82	105.14 ± 12.68	67.45 ± 9.01	1.45 ± 0.26	16.88 ± 2.71	0.31 ± 0.05	82.95 ± 20.66	92.90 ± 3.00
Weaning failure group	18	10.6 ± 4.57	9.00 ± 4.39	2.67 ± 2.09	196.33 ± 9.48	103.78 ± 89.48	64.89 ± 9.29	1.02 ± 0.11	21.54 ± 2.76	0.21 ± 0.02	109.34 ± 18.10	90.33 ± 3.96
*t* value		0.211	−0.348	1.556	2.743	0.409	1.001	6.864	−6.06	7.559	−4.698	2.757
*P* value		0.834	0.729	0.125	0.008	0.684	0.321	<0.001	<0.001	<0.001	<0.001	0.008

### Collinearity diagnosis and multivariate binary logistic regression

There was a linear relationship between continuous independent variables and the log-transformed values of the dependent variable. Among the independent variables, there was multicollinearity between DE and DE-RSBI, as well as between DTF and DTF-RSBI. DE-RSBI and DTF-RSBI were calculated based on DE and DTF, respectively, so DE and DTF were excluded for further analysis. There were no outliers. Logistic stepwise regression analysis showed that P/F ratio, DE-RSBI, DTF-RSBI, and PCIS were independently associated with weaning success. The combined predictive model showed a high goodness of fit (*P* = 0.967), and all regression coefficients (b) were statistically significant. The model equation is expressed as Logit*P* = 73.299–0.253 P/F ratio + 0.525 DE-RSBI + 0.055 DTF-RSBI-0.43 PCIS, as shown in Table 2 and Table 3.

**Table 2:** Logistic regression univariate analysis of independent variables and logit transformation of dependent variables, and multicollinearity test among independent variables.

Independent variable	Linearity between independent variable and logit-transformed dependent variable		Multicollinearity among independent variables	
	Wald value	*P* value	Tolerance	Variance inflation factor
P/F ratio	0	0.99	0.852	1.174
DE-RSBI	0.183	0.669	0.75	1.333
DTF-RSBI	0.943	0.331	0.727	1.376
PCIS	0.698	0.403	0.857	1.167

**Table 3:** Multivariate binary logistic regression analysis.

Variable	*B*	*SE*	Wald *χ*^2^ value	*P* value	OR value (95% CI)
P/F ratio	−0.253	0.1	6.430	0.011	0.777 (0.639, 0.944)
DE-RSBI	0.525	0.187	7.846	0.005	1.691 (1.171, 2.441)
DTF-RSBI	0.055	0.027	4.078	0.043	1.056 (1.002, 1.114)
PCIS	−0.43	0.205	4.392	0.036	0.650 (0.435, 0.972)

### Predictive performance of P/F ratio, DE-RSBI, DTF-RSBI, PCIS, and the combined prediction model for weaning

**Figure 1:**
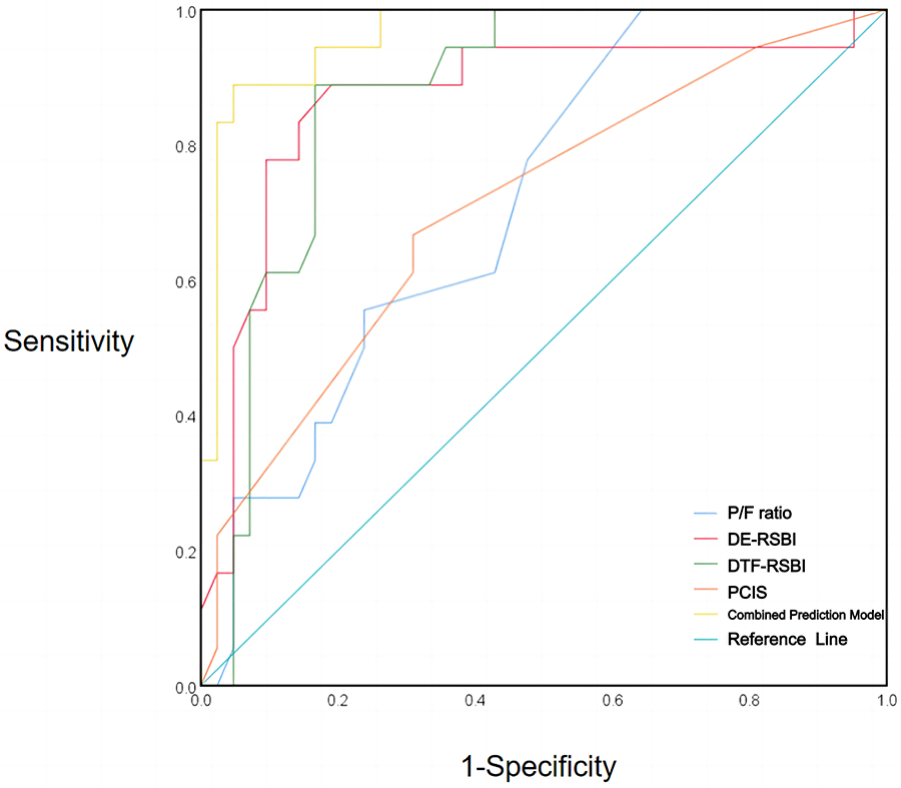
ROC curves of P/F ratio, DE-RSBI, DTF-RSBI, and PCIS for extubation prediction alone and in combination.

**Figure 2:**
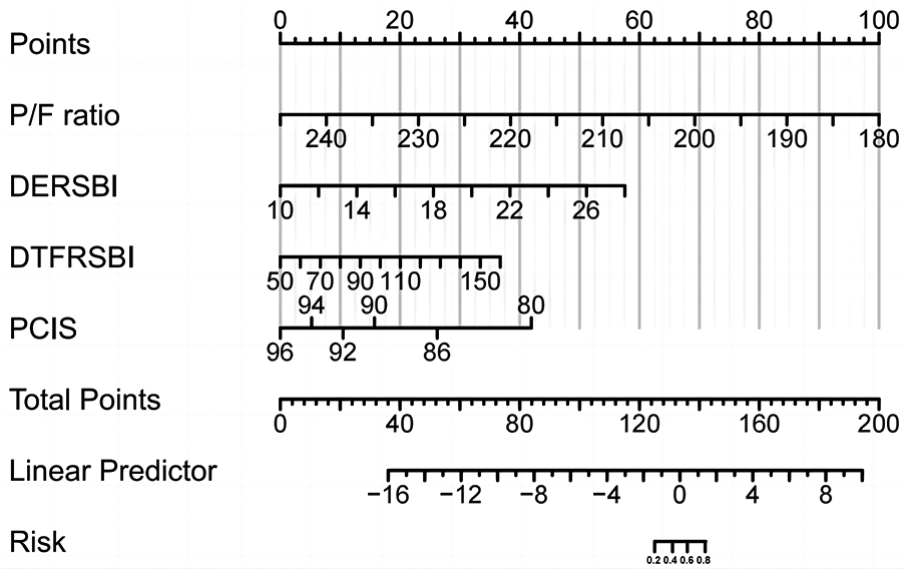
Risk nomogram conducted by logistic regression.

In the weaning success group, P/F ratio, DE-RSBI, DTF-RSBI, PCIS, and the combined predictive model had sensitivities and specificities of 76.2%, 88.9%, 88.9%, 69.0%, 89.7%, and 66.7%, 81.0%, 85.7%, 66.7%, 95.2%, respectively. The AUC values for P/F ratio, DE-RSBI, DTF-RSBI, PCIS, and the combined prediction model were 0.749, 0.853, 0.880, 0.700, and 0.960, respectively. The AUC of the combined prediction model was significantly greater than the AUC of each single indicator (*Z* = 9.129, 2.061, 2.075, 8.326, *P* < 0.05), as shown in Table 4 and Figure 1. The nomogram of the combined prediction model is shown in Figure 2.

**Table 4:** JP/F rationt predictive performance of the P/F ratio, DE-RSBI, DTF-RSBI, PCIS, and the combined predictive model for aircraft retrieval.

Index	AUC	95% CI	Sensitivity (%)	Specificity (%)	Cut off value	*P* value
P/F ratio	0.749	(0.624, 0.875)	76.2	66.7	199.5	<0.01
DE-RSBI	0.853	(0.735, 0.970)	88.9	81.0	19.55	<0.01
DTF-RSBI	0.880	(0.792, 0.968)	88.9	85.7	90.00	<0.01
PCIS	0.700	(0.554, 0.846)	69.0	66.7	93.0	0.015
Combined predictive model	0.960	(0.915, 1.000)	88.9	95.2	0.511	<0.01

## Discussion

According to relevant studies (8, 9), approximately 30% of children in the PICU require mechanical ventilation treatment for approximately one week, and 20%–23% of mechanically ventilated children may face the risk of weaning failure. Weaning failure has been a hot topic and a challenging issue for PICU physicians. Premature weaning may increase the burden on the respiratory and cardiovascular systems of children, leading to weaning failure and is an independent risk factor for increased mortality in mechanically ventilated children. However, unnecessarily prolonging mechanical ventilation not only increases healthcare costs but also elevates the risk of ventilator-associated complications (10, 11). Therefore, by analyzing factors influencing weaning and exploring reasonable predictors of weaning, clinicians can better determine the timing of weaning and improve its success rate, ultimately enhancing healthcare quality and reducing resource wastage.

Studies have shown a close association between diaphragmatic injury caused by mechanical ventilation and weaning failure (12, 13). Diaphragmatic ultrasound is an effective tool to assist physicians in assessing diaphragmatic injury. Parameters such as DE and DTF are highly correlated with diaphragmatic function assessment and can be used to predict weaning outcomes. Research by En-Pei Leefound a significant decrease in DE and DTF within the first 24 h of mechanical ventilation in 31 pediatric patients (14). After extubation, there was a significant difference in DTF between successful and unsuccessful extubation groups, with DTF <17% showing a correlation with extubation failure. Mistri S found that children experience progressive diaphragmatic atrophy after mechanical ventilation (15). Yang discovered that there was no statistically significant difference in DE between the weaning success and failure groups, but DTF, as a predictive indicator, had a sensitivity of 82%, specificity of 81%, and an AUC of 0.89, making it a good predictor (16). Research by Fossat G found that the ratio of RSBI and RSBI/DE could not predict weaning success, but combining ultrasound DE and DTF as predictive indicators might be worth further investigation (17). The study by Song J revealed that the sensitivity of DE-RSBI is 89.2%, specificity is 56.9%, with an AUC of 0.813.While DTF-RSBI shows a sensitivity of 67.6%, specificity of 93.2%, with an AUC of 0.859 (18). Despite the limited research on the ultrasound indicators of diaphragm function, DE-RSBI and DTF-RSBI, in predicting extubation outcomes in mechanically ventilated pediatric patients, existing studies have confirmed that high DE-RSBI and DTF-RSBI are independent risk factors for extubation failure in adults (19). DE-RSBI and DTF-RSBI have also been shown to have diagnostic efficacy superior to a single rapid shallow breathing index. These studies confirm the feasibility of using DE-RSBI and DTF-RSBI to predict extubation outcomes, providing a theoretical basis for our research. Our study further demonstrated that although DE and DTF are independent risk factors for extubation failure, they exhibit significant collinearity with DE-RSBI and DTF-RSBI. Therefore, our study retained DE-RSBI and DTF-RSBI as independent risk factors for extubation failure and conducted ROC curve analysis, with DE-RSBI showing a sensitivity of 88.9%, specificity of 81.0%, and an AUC of 0.853, and DTF-RSBI showing a sensitivity of 88.9%, specificity of 85.7%, and an AUC of 0.88. Importantly, the cutoff values for DE-RSBI and DTF-RSBI in children are significantly higher than in adults due to the higher respiratory rate in children and lower DE and DTF compared to adults. Therefore, ultrasound measurement of diaphragm parameters DE-RSBI and DTF-RSBI can provide guidance for the selection of the optimal timing for clinical extubation, thus improving the prognosis of pediatric patients.

Dynamic monitoring of the P/F ratio can effectively reflect the body's oxygen deficiency status and pulmonary ventilation and gas exchange function, making it widely used in clinical practice (20). The study by Sunitha Palanidurai found that when PEEP is greater than 5 mmHg, the area under the ROC curve for predicting extubation outcomes using the P/F ratio is 0.659. When using 135 mmHg as the threshold, the sensitivity and specificity for predicting extubation are 59.5% and 65.5%, respectively (21). However, this study found that P/F ratio had a sensitivity and specificity of 76.2% and 66.7%, respectively, with an AUC of 0.749, which is slightly lower than the previous results. This discrepancy may be due to differences in the study population. Combined with the common occurrence of multi-organ dysfunction in critically ill children with ARDS, using P/F ratio as a single predictor of weaning outcomes may lack sensitivity and specificity.

The PCIS is a scoring system used to assess the severity of illness in children. It helps physicians accurately assess the child's condition, formulate treatment plans, and effectively prevent and treat complications, thereby improving the child's prognosis. Lower PCIS indicate a more severe condition and may be associated with a higher risk and greater degree of multi-organ damage. Multi-organ dysfunction is a major cause of death in critically ill children, and PCIS can reflect the degree of organ damage and the risk of death to varying degrees. Fang C found that as the PCIS upon admission decreased, the proportion of mechanically ventilated children and the duration of mechanical ventilation increased (22). Critically ill children upon admission have a higher likelihood of respiratory system involvement and an increased likelihood of requiring mechanical ventilation due to hypoxia. However, this study found that PCIS had limited accuracy as a single predictor, with a sensitivity and specificity of 69.0% and 66.7%, respectively, and an AUC of 0.7.

Compared to predicting outcomes based on a single indicator, combined prediction can improve sensitivity and specificity to some extent. This study's logistic regression analysis revealed that P/F ratio, DE-RSBI, DTF-RSBI, and PCIS were all independent risk factors for pediatric mechanical ventilation weaning. The combined prediction model, P/F ratio, DE-RSBI, DTF-RSBI, and PCIS, all demonstrated a certain predictive performance for weaning success. The combined indicator's sensitivity for predicting pediatric mechanical ventilation weaning success was 88.9%, with a specificity of 95.2% and an AUC of 0.960. Compared to using P/F ratio, DE-RSBI, DTF-RSBI, or PCIS individually, the combined prediction model showed higher predictive capabilities, surpassing the overall diagnostic performance when any single indicator was used. It can be widely applied in clinical practice to facilitate timely and accurate weaning, reducing the risk of severe weaning-related complications.

Identifying factors affecting weaning outcomes via diaphragm ultrasound is crucial, yet causal relationships are often unknown. Causal inference methods can illuminate these connections, aiding clinical management (23). Predictive models highlight factors like clinical characteristics and disease status, but fail to establish causality. Causal inference involves accounting for confounders like underlying diseases and treatments to ensure accuracy. Challenges include temporal precedence and unobserved confounders, addressed with advanced statistical methods. In further research, causal inference will be conducted, as it provides deeper insights, guiding treatment decisions and resource allocation, thus significantly impacting clinical practice. Additionally, multicenter studies and external validation will be conducted.

## Conclusions

P/F ratio, DE-RSBI, DTF-RSBI, and PCIS are independent risk factors for pediatric weaning from mechanical ventilation. While individual diaphragmatic ultrasound parameters hold value, a combined prediction model using P/F ratio, DE-RSBI, DTF-RSBI, and PCIS offers higher predictive value for pediatric weaning.

## Data Availability

The original contributions presented in the study are included in the article/Supplementary Material, further inquiries can be directed to the corresponding author.
